# Delivery of iron-fortified yoghurt, through a dairy value chain program, increases hemoglobin concentration among children 24 to 59 months old in Northern Senegal: A cluster-randomized control trial

**DOI:** 10.1371/journal.pone.0172198

**Published:** 2017-02-28

**Authors:** Agnes Le Port, Tanguy Bernard, Melissa Hidrobo, Ousmane Birba, Rahul Rawat, Marie T. Ruel

**Affiliations:** 1 Poverty Health and Nutrition Division, International Food Policy Research Institute, Dakar, Senegal; 2 Markets, Trade and Institutions, International Food Policy Research Institute, Dakar, Senegal; 3 GREThA, UMR CNRS 5113, University of Bordeaux, Bordeaux, France; 4 Poverty Health and Nutrition Division, International Food Policy Research Institute, Washington DC, United States of America; TNO, NETHERLANDS

## Abstract

**Background:**

Innovative strategies are needed to enhance the nutritional impact of agriculture. Value chain approaches, which use supply chains to add value (usually economic) to products as they move from producers to consumers, can be used to increase access to nutritious foods and improve nutritional status. This study tested whether a dairy value chain could be used to distribute a micronutrient-fortified yoghurt (MNFY) (conditional upon the producer supplying a minimum amount of cow milk/day) to improve hemoglobin and reduce anemia among preschool children in a remote area in Northern Senegal.

**Methods:**

A cluster randomized control trial was used to compare 204 children (24 to 59 months of age at baseline) from households who received the MNFY coupled to a behavior change communication (BCC) campaign focusing on anemia prevention to 245 children from a control group (receiving BCC only) after one year. Randomization was done at the level of the family concession (households from the same family) (n = 321). Eligible households had a child of the target age and were willing to deliver milk to the dairy factory. Changes in anemia and hemoglobin between groups were assessed using mixed regression models.

**Key findings:**

Anemia prevalence was very high at baseline (80%) and dropped to close to 60% at endline, with no differences between intervention groups. Hemoglobin increased by 0.55 g/dL, 95%CI (0.27; 0.84) more in the intervention compared to the control group after one year, in models that controlled for potentially confounding factors. The impact was greater (0.72 g/dL, 95%CI (0.34; 1.12)) for boys, compared to girls (0.38 g/dL, 95%CI (-0.03; 0.80)).

**Conclusion:**

The dairy value chain was a successful strategy to distribute MNFY among pastoralists in Northern Senegal, and increase Hb concentrations among their children. This study is one of the first proofs of concept showing that a nutrition-sensitive agriculture value chain approach can contribute to improved child nutrition in a remote pastoralist population.

**Trial registration:**

ClinicalTrials.gov NCT02079961

## Introduction

Micronutrient deficiencies including iron, iodine, folate, vitamin A and zinc are the most widespread global nutrition problem, affecting more than 2 billion people worldwide [1]. Anemia, of which globally about half is due to iron-deficiency [2], affects women of reproductive age and young children who are particularly vulnerable due to their high physiological requirements and for young children, given their limited ability to consume sufficient amounts of high-iron foods. In Senegal, the prevalence of anemia is extremely high, with 76% of children under-five years of age being anemic [3]. Causes of anemia are multifactorial and include nutritional deficiencies, infections, and blood disorders.

Interventions to prevent and treat iron-deficiency anemia include supplementation, food fortification, and dietary modification. The primary platforms for delivering micronutrient supplements, fortified foods, or home fortification products like micronutrient powders (MNP) and lipid-based nutrient supplements (LNS) are through the health system, using either facility-based or community outreach strategies [4]. In Senegal, commercially produced complementary foods like fortified infants cereals are available, but they are usually unaffordable for resource-poor and remote populations who are likely to need them the most [5]. Given resource and capacity constraints in health systems in developing countries, and in Africa in particular, and the limited purchasing power of vulnerable populations, alternative or complementary interventions and delivery strategies are needed to address iron-deficiency anemia.

One novel potential delivery strategy for micronutrient interventions is through a value chain approach. A value chain is a supply chain in which value (usually economic) is added to the product as it moves from producers, to processors and intermediaries, to final consumers. A nutrition sensitive value chain focuses on integrating nutritional objectives and interventions in the supply chain, without compromising the product’s economic value, taking into account the nutritional needs of the multiple actors, including the consumers it supplies [6]. Value chain interventions that focus on food fortification need to identify the proper vehicle (food) and fortificant(s), accounting for a series of factors such as bioavailability, interaction with other foods in the diet, availability, acceptability, and cost. Other important considerations include clearly specifying the target population and ensuring quality of the final product and appropriate demand (e.g. ensuring that the quantity usually consumed by the target population is sufficient to improve status in a sustainable manner). In order to accomplish these aims, a strong behavior change communication (BCC) strategy to stimulate demand and improve health and nutrition practices at the consumer level is needed, along with an approach to ensure ready access to a sufficient supply of products by the target population [7].

This paper reports on a pilot study of a nutrition sensitive dairy value chain conducted in Richard Toll, in the North-East of Senegal, in partnership with a local dairy factory (La Laiterie du Berger, LDB) and its network of dairy farmers. The study incorporated the distribution of a micronutrient fortified yogurt (MNFY) produced from milk supplied by the participating dairy farmers, and used as an incentive to increase milk delivery and to improve children’s nutritional status (with a focus on anemia). Milk producers who met a fixed quantity of milk delivered five days a week received daily MNFY the following week and were instructed to give it to their children 24 to 59 months of age. The MNFY product was delivered at collection points that were easily accessible to women, thus targeting women as the main recipient. In addition, a BCC strategy aimed at improving infant and young child feeding practices, including the use of micronutrient-rich food or fortified products, was launched in the whole study area.

This project used a cluster randomized control trial to test two main objectives. The first objective was to assess whether the MNFY had a positive impact on milk production (regularity and quantity) throughout the year, and most importantly during the dry season [8]. The second objective was to test whether the MNFY distributed through the dairy value chain and combined with the BCC strategy had an impact on improving hemoglobin (Hb) and reducing anemia among children in dairy farmer households, compared to children from a control group that received the BCC only. This paper present results on the second objective.

## Materials and methods

### Study area

Richard Toll is located in the northern Saint-Louis region of Senegal, bordering Mauritania and the Senegal River. The district extends into a vast arid area where access to health centers is limited. In the region of Saint-Louis, the average distance to reach a health post is 7 km. Only 19 health posts and one hospital were available at the time of the study in the district of Richard Toll [9]. BCC activities on nutrition and health at the community level are part of the curriculum of the Cellule de Lutte Contre la Malnutrition (CLM), the agency in charge of technical assistance in defining and implementing the national policy in nutrition, called the Programme de Renforcement de la Nutrition (PRN) (Nutrition Strengthening Program). However, BCC activities were not yet implemented in the study area in 2012.

The local dairy factory (LDB) was set up in Richard Toll in 2006, and based its activity on a system of milk collection from semi-nomadic pastoralists. Pastoralists belong to the semi-nomadic ethnic-group of Pulaar (Fulanis) who live in concessions (extended family unit) and migrate with their herds every year during the dry season (from November to June) when local pastures are no-longer sufficient for animal grazing. Due to insufficient water and pasture, milk production is at its lowest during the dry season. For the Pulaar, gender roles in milk production are clearly established at a young age, with women being in charge of milk production, and men of herd management [10].

### Intervention description

The LDB collects milk from each producer twice a day through a network of 94 collection points close to their home. Milk collection trucks follow four 50 km routes throughout the area, collect the milk, and bring it back to the factory for processing and production of non-fortified yoghurt usually sold in urban centers. For this study, a formal contract was signed between the LDB and the pastoralists, whereby producers committed to supply 0.5 L of milk per lactating cow per day, 5 days per week. Households in the intervention group who fulfilled the contract in the previous week received one sachet of MNFY per child 24–59 months of age per day for seven consecutive days, in addition to the normal payment of 200 FCFA per liter of milk delivered. Households in the control group who fulfilled the contract received the normal payment for each liter of milk delivered but no MNFY. MNFY sachets were delivered daily to collection points easily accessible to women by the same truck that collected milk from the suppliers, thereby saving on transport/delivery costs. The MNFY was produced specifically for the study, using the milk collected from the pastoralists. The 80 g sachet of yoghurt was mixed with grains of millet (recipe of a traditional Senegalese yoghurt) and fortified with 2.1 mg of iron-EDTA (in addition to 2.25 mg of zinc, 24 μg of Iodine and 120 μg of Vitamin A). Implementation of the program was conducted by the Gret, an international NGO, working closely with the milk factory and its milk suppliers. The target age range for children in the intervention (24 to 59 months) was agreed upon among all actors involved in the intervention; children beyond the first 1,000 days were selected because of the dairy factory’s’ concerns regarding authorizations and infrastructures needed to produce complementary foods for infants and young children less than 24 months of age. The micronutrient formulation of the MNFY was therefore tailored to the needs of children 24–59 months of age.

In addition, starting 3 months after the beginning of the intervention and continuing for 9 months, the CLM launched a BCC intervention implemented by two local non-governmental organizations (NGO) in both intervention and control areas. The BCC strategy included group sessions organized once a month in each village, home visits for households located in remote hamlets, social mobilization activities (events such as theater plays presented in big villages twice during the one-year study), and nine radio spots. Messaging focused on essential nutrition actions (ENA) [11] including optimal infant and young child nutrition during their first 24 months of life, the importance of micronutrients, the role of dietary diversity, the importance of consuming iron-rich, or fortified foods to prevent anemia, and the identification of symptoms of anemia. The BCC activities did not provide any information on the MNFY itself, because they were part of the standard PRN program of community based BCC implemented nationwide, and thus it was intended to increase demand for any fortified product.

Given the slight delay in rolling out the BCC strategy and the consequences of seasonality and migration on milk production, the intervention was implemented slightly differently during three periods: 1) February-March 2013 (dry season): MNFY was distributed, as planned, in the intervention group conditional on households providing 0.5L of milk/per cow, with no BCC in either intervention or control group; 2) April-June 2013 (peak dry season): MNFY was distributed in the intervention group and BCC in both groups; however starting in mid-May, the conditionality for milk provided was decreased purposively to 0.3 L of milk per lactating cow per day, 5 days per week, because implementers realized that the cut-off used previously was too difficult to achieve, especially during the dry season; 3) July-December 2013 (rainy season until November): MNFY was distributed in intervention group conditional on households providing 0.3L of milk/per cow with BCC implemented in both intervention and control group.

### Evaluation design and randomization

The evaluation used a cluster randomized control trial to compare the Hb concentration and anemia prevalence of children aged 24 to 59 months old in two groups: intervention and control. Concessions, which are units of 3–7 households usually related to each other, were randomly assigned to intervention and control group. In December 2012, 16 public gatherings were organized along the four milk collections routes by the implementing partner Gret to inform suppliers who were selling milk to the milk factory at the time, or those who had in the past and were willing to re-engage, about the objectives of the study, the milk production contract, the conditions of the MNFY delivery, the biological and other data collected, and the possibility to withdraw the study at any time. An information sheet was also distributed. The same day, public lotteries were performed to assign concessions to the intervention or control group. Suppliers were invited to meet at several points of the milking routes. Taking into consideration the context of the project area and for logistical reasons, we stratified the randomization of concessions (n = 321) at the lottery level per milking route level, in order to guarantee that, within each stratum or milking route, each of the intervention arms was represented equally. All the suppliers’ names were written on small pieces of paper, stapled by concession, collected into a barrel, and selected at random (publicly) to allocate concessions to intervention and control group. Eligibility criteria for households of active milk suppliers were that: 1) they agreed to participate in the study and 2) they had at least one child within the target age of 24–59 months. Households who accepted to participate in the study and declared having children aged 24–59 months old living in their household were asked to sign a contract. To avoid sharing of the MNFY with non-target children, the project provided MNFY to all children 24–59 mo old in the target concessions, even if they did not have a contract with the milk factory. Households from the intervention and control groups were paid 200 CFA/liter monthly for the milk they delivered to the dairy factory.

### Sample size calculations

We estimated that daily consumption of MNFY by target children would reduce the prevalence of anemia by 15 percentage points (pp) at endline from an initial level of 75%. With a power of 80%, 321 clusters randomized, an anemia intra cluster correlation (ICC) set at 0.3, a one-sided test, and equal cluster sizes assumed, we estimated, using sampsi/sampclus command in Stata, that a total of 133 children aged 24–59 months per group were needed to detect a 15 pp difference between interventions groups at endline. Taking into account a potential 20% loss of follow-up and 20% of households not meeting the criteria for receiving the incentive, we estimated that we would need to enroll 186 children per group. We estimated that 160 clusters per group would allow to detect a difference of 0.43 g/dL in hemoglobin concentration from an initial hemoglobin concentration at 11.5 g/dL (SD = 0.17)[3].

### Study enrolment at baseline and timing of assessments

The main criteria for household enrolment was to have at least one child 24–59 months of age living in the household at the time of the baseline survey, and having committed to fulfil the contract of milk delivery. Written informed consent was obtained from all survey respondents (household head and spouse). A contact sheet was left with each household as well as copies of informed consent sheets which were also stored by the survey team. The baseline survey was conducted in January 2013 and the endline survey in January 2014, with two additional follow-up surveys in April 2013 (follow-up 1, F1) and in September 2013 (follow-up 2, F2). At endline, children followed-up were 36–71 months.

### Data collection and outcomes

Data were collected using questionnaires at the household, maternal and child level, during the baseline and endline surveys. Maternal questionnaires were administered on all mothers in a household with children 24–59 months of age at baseline, which given polygamy and household structure meant that some households had more than one mother questionnaire administered. At baseline, heads of household were asked about household characteristics such as household composition, cattle ownership, ownership of assets, and household food security using the HFIAS score[12].

Maternal knowledge was assessed through a questionnaire asking mothers to list iron-rich foods and the consequences of anemia in children. Mothers were considered knowledgeable if they were able to mention at least 2 out of 3 iron-rich foods (i.e. liver, fish or meat) and at least 2 out of 5 anemia consequences (i.e. difficulty at school, impaired development, slow growth, weak immunity defenses, and fatigue).

Data on availability of the MNFY at the household level were collected at the two follow up surveys (F1 at 3 months; and F2 at 8 months following baseline) and at endline, through maternal recall. Mothers were asked if children from their household had consumed the MNFY, in the 3 days prior to the interview.

Data on anemia (the study’s primary outcome) was collected at all four data points (baseline, F1, F2 and endline) using Hb concentration measured through a finger prick using a Hemocue device 201+ (HemoCue Ltd, United Kingdom). HemoCue devices 201+ were cleaned and checked for accuracy prior each data collection, using blood samples to check for repeatability. Anemia was defined as Hb below 11 g/dL and severe anemia, as Hb below 7g/dL. Children who were severely anemic and/or detected as suffering from severe acute malnutrition at any of the data collection points were referred to the closest health center. Mothers were given a small fee for transportation. Basic treatment (iron treatment, antiparasitic treatment, antibiotics) was provided at no cost to the mother or the health center, conditional on presenting the project’s referral letter. Health centers were reimbursed by the survey managers for treatment costs after the surveys and data on children were collected. Data were collected using computer assisted personal interviews (CAPI) using CSPro software.

### Deviations from the initial study protocol

In the initial study protocol, the intervention was planned to last 6 months, using a cross-sectional design, with a baseline survey in January 2013, an endline survey at 6 months and a post-intervention assessment at 12 months. Before the start of the program, a census was organized in the study area to interview the 700 milk suppliers of the LDB on their willingness to participate in the program and to verify the number of children of target age living in their households. At the time of the census, all supplier households reported having at least one child between 24 and 59 months of age with an average of two children in this age range, suggesting a total number of eligible children of 1400. However, when enumerators assessed the children’s exact age during the baseline survey, only 310 of these households, from 247 concessions (instead of 320), were found eligible (with a total of 462 children aged 24 to 59 months old). With 247 concessions, the sample needed to detect a 15 pp difference of anemia prevalence between interventions groups at endline with a power at 80% was 346 children in total (~ 173 per arm). Adding 20% of potential loss of follow-up and 20% of households not meeting the criteria for receiving the incentive, we needed 484 children in total (~ 242 per arm), slightly more than the estimated number of eligible children in the population sampled (462 children in total (~ 231 per arm)). Given this slightly lower sample size than expected, the study team decided to move from a cross-sectional design to a longitudinal design, which followed children over time from baseline to endline.

The program also faced some delays in implementation, as noted above. The MNFY distribution started in February (instead of January) and the BCC campaign in April. Given these delays, the duration of the program was extended by 6 months, until December 2013 (instead of June), and measurements were taken at baseline (January 2013), follow-up 1 (F1) in April 2013, follow up 2 (F2) in September 2013, and at endline in January 2014.

The initial protocol for this study and supporting CONSORT checklist are available as supporting information; see S1 Consort Checklist and S1 Protocol.

### Statistical methods

Comparison of baseline characteristics between intervention and control group was conducted to assess the quality of randomization in bivariate analysis, linear mixed-effect regression models using random effect at the concession level (cluster) for continuous variables and for binary variables (linear probability model) and using clustered Chi-squared tests for categorical variables (stata command clchi2). Comparison of anemia and severe anemia prevalence between intervention groups and boys and girls, percentage of children surveyed at different time-point by intervention group, maternal knowledge and household level of MNFY consumption between boys and girls were also done using linear mixed-effect regression using random effect at the concession level (cluster).

The impact analysis was performed using intent to treat estimates, taking into account the cluster design and a level of statistical significance of 5% for a 1-sided test. We compared main outcomes by intervention group using linear mixed-effects regression models with random effects at two levels—random intercept and random slope at the child level and random intercept at the cluster level of concession. We found that adding time as a random slope improved the fit of the model significantly using a likelihood ratio test. We used an unstructured covariance for the random effects at the child level, after comparing models with different covariance structures using the Akaike information criterion.

Models for Hb and anemia included all children having a baseline measure of Hb, regardless of whether or not they were present at any follow up measurement, mixed models allowing missing data for repeated measures. Interactions terms (Intervention X Time) were used to assess the effect of the intervention on Hb and anemia (Intervention vs control) between baseline and different time points (F1, F2, endline) (baseline serving as reference). Models were run separately by gender to take into account potential differences between boys and girls related to health or migration. Models controlled for the following potential confounding factors: wealth index, size of the household, number of lactating cows (as a wealth proxy), maternal age, children’s age, sex, and iron treatment for severe anemia at baseline (effective visit at the health center).

A wealth index was established for each household, using multiple component analysis (mca stata command). The creation of the wealth index was based on the ownership of 27 assets (household assets: benches, tables, seats, beds, jars/calabashes, pestles/mortars, mattresses, radios, watches, chairs/Living-room, TV, mobile phones, fridges, carts, bicycles, motorcycles, cars; agriculture equipment: sickles, seed drills, spades, traditional hoes, other hoes, machetes/axe; animals: poultries, lambs, goats, donkeys). The two first principal components explained 78.71% of the total variance and were used in the analysis. All data analysis was performed using STATA 14.0 software.

### Ethical approval

Ethical approval for this survey was obtained from the National Committee for Ethics and Research for Development for one year, under the protocol number SEN12/57 and from the International Food Policy Research Institute Institutional Review Board (IFPRI IRB). An amendment of the protocol was submitted to both the National Committee of Senegal and the IFPRI IRB to include an additional measure of hemoglobin in April 2013. The protocol was registered in the clinical trial.gov website (NCT02079961).

## Results

### Study population

**Fig 1:**
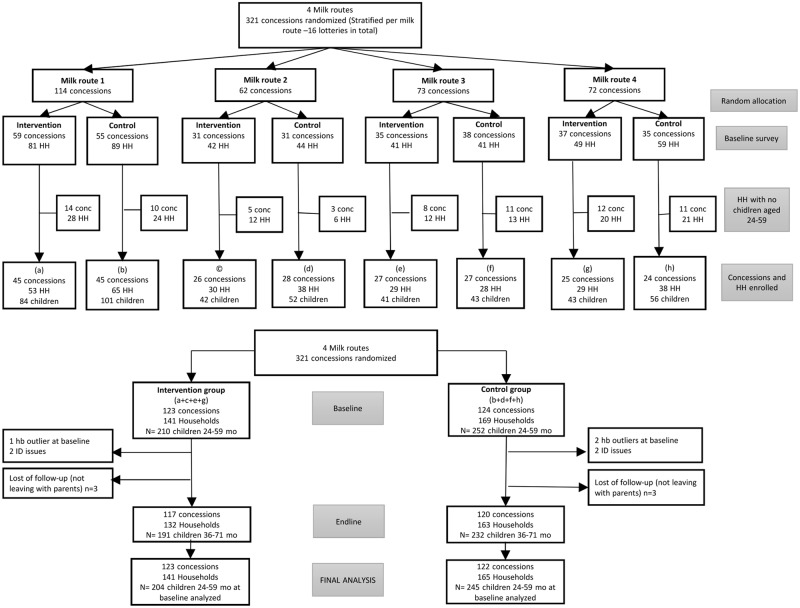
Flow chart, dairy value chain in Senegal, Richard toll, 2013–2014.

A total of 321 concessions were randomized to the two study groups during public lotteries, 162 in the intervention group and 159 in the control group. A total of 446 households were surveyed at baseline, with 213 in the intervention group and 233 in the control group (Fig 1). However, only 310 households (from 247 concessions) had children of the target age after age estimation was performed by enumerators when no official document indicating date of birth was available. This led to a baseline sample of 462 children (210 in intervention; 252 in control group) from 310 households in 247 concessions. Among households with a child of the target age at baseline, 3 children in the intervention group and 4 children in the control group did not have a valid baseline Hb measurement. Reasons included children identification issues and technical issue with Hb outlier. In addition, 6 children (3 in intervention; 3 in control group) were not living with their parents at any follow-up survey and considered lost-of follow-up. During follow-up surveys (F1 in April 2013 and F2 in September 2013), cattle migration from these semi-nomadic pastoralists led to absences. However, all children were kept in the analysis even if they were absent during one or more follow-up visits (82% of children present at F1, 84% at F2, balanced proportion of children present between intervention and control groups). At endline, 423 children aged 36–71 months old were present (94% of the initial sample), with 191 children in the intervention group (94%) and 232 in the control group (95%). For final analysis, of the 306 households analyzed across 391 mothers, 204 children were in the MNYF group and 245 were in the control group.

### Baseline characteristics

The study population is characterized by a high level of food insecurity, with 55% of households being severely food insecure and 28% moderately food insecure at baseline. Access to the health services is very low, with 61% of mothers interviewed having delivered at home, only 24% having vaccination cards for their children, and 14% mentioning that their children had been dewormed in a health center during the past 2 months. The groups did not differ at baseline in household and maternal characteristics (Table 1). Households had a low (~ 9) mean HFIAS score on a 0–27 scale. Mothers were 29 years old on average and children 41 months old at baseline; 51% of children were boys.

**Table 1:** Baseline characteristics in intervention group (MNFY+BCC) and control group (BCC).

	All	Intervention	Control	p
**Concession characteristics (N)**	247	123	124	
Number of HH per concession	1.25 (0.62)	1.15 (0.45)	1.35 (0.74)	0.01^a^
Number of child per concession	1.83 (1.09)	1.66 (0.86)	2.01 (1.25)	0.01 ^a^
**Household characteristics (N)**	306	141	165	
Size of the household (number of members)	9.63 (4.17)	9.87 (4.52)	9.44 (3.86)	0.47 ^b^
Number of lactating cows	6.64 (3.58)	6.79 (3.96)	6.51 (3.23)	0.23 ^b^
Wealth index (0–1)	0.61 (0.20)	0.62 (0.19)	0.61 (0.20)	0.89 ^b^
Households (%)				
1st tertile Wealth index	0.36	0.32	0.39	
2d tertile Wealth index	0.34	0.38	0.31	
3rd tertile Wealth index	0.30	0.30	0.30	0.46 ^c^
Mean HFIAS score (0–27)	8.93 (5.82)	8.95 (5.57)	9.91 (6.04)	0.21 ^b^
Households (%)				
Food secure	0.09	0.07	0.11	
Mildly food insecure	0.07	0.06	0.08	
Moderately food insecure	0.28	0.33	0.24	
Severely food insecure	0.55	0.54	0.56	0.28 ^c^
**Maternal characteristics (N)**	391	183	208	
Mother’s age (years)	29.08 (8.82)	28.75 (9.09)	29.34 (9.37)	0.59 ^b^
Mother’s BMI (kg/m2)	21.81 (3.32)	21.90 (3.35)	21.91 (3.42)	0.83 ^b^
**Children characteristics (N)**	449	204	245	
Age, (months)	37.57 (10.24)	37.68 (10.40)	37.48 (10.13)	0.83 ^b^
Sex, % male	0.51	0.50	0.53	0.58 ^c^

^a^ p-values obtained using t-test.

^b^p-values obtained with mixed linear regression testing the difference between study groups, controlling for clustering (random effect) at the concession level.

^c^ p-values obtained from a chi-square test controlling for clustering by using clustered Chi-squared test in Stata.

Values are means (SD) or percentages.

Abbreviations: BCC: Behavior change communication campaign; BMI: Body Mass Index; HFIAS: household food security access scale; HH: Households; MNFY: micronutrient fortified yogurt;

### Contract fulfillment and exposure to MNFY

**Fig 2:**
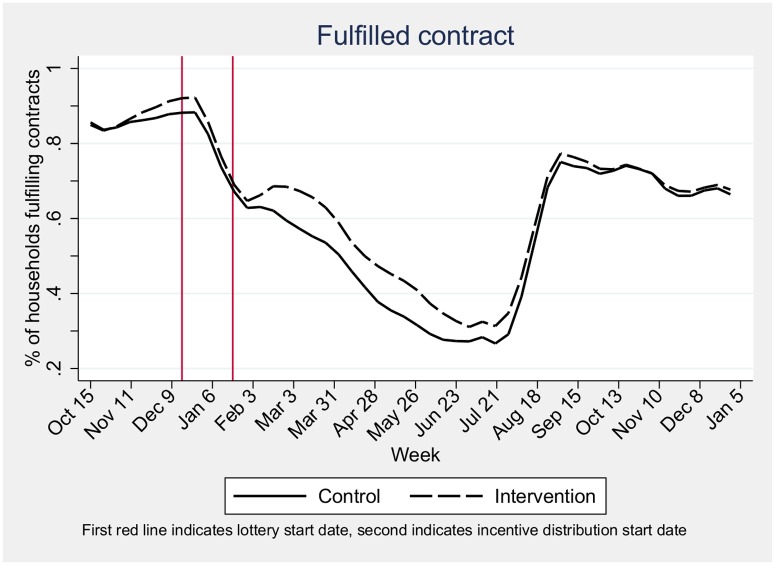
Impact of the intervention on household delivering milk by intervention group, Richard Toll, 2013–2014.

The distribution of MNFY depended on households satisfying the contract of 0.5L (and later 0.3L) of milk/cow per day for 5 days in a given week. Fulfillment of contracts was high at the start of the intervention (80% in January 2013), before it dropped significantly to 27% at the height of the dry season in June for control households and 31% in intervention households (Fig 2). In August, with the first rain, contract fulfillment spiked again, reaching approximately 75% for both intervention and control groups. Contract fulfillment remained high for the remainder of the study, at around 70% in January 2014.

The percentage of households in the intervention group whose children had consumed the MNFY in the past 3 days, based on maternal recall, was 63% at F1, 87% at F2 and 60% at endline (S1 Table). At F1, 66% of boys vs 60% of girls of target age lived in a HH receiving MNFY; at F2 and endline respectively, the percentages were 86% and 61% among boys, vs 85% and 63% among girls. There were no statistically significant gender differences on HH receiving MNYF at any point in time. In the control group, 4% (5/139) of mothers mentioned children in their households consuming MNFY during the 3 days prior F2 (no available information for F1) and 9% prior to endline (14/161).

### Seasonal migration and missing data

S2 Table shows the number of children surveyed at different time points, disaggregated by intervention group and child gender. Only 6% of children of children included in the analysis at baseline were not surveyed at endline, with no difference between intervention and control group (6% and 5% respectively), or gender (7% vs 6% among boys, 6% vs 5% among girls). The proportion of children not surveyed (missing data in analysis) at F1 and F2—mainly due to seasonal migration—were larger (18% and 16% respectively), but did not differ between intervention and control groups, nor between boys and girls at the different data collection points.

### Hemoglobin concentration and anemia prevalence at baseline, F1, F2 and endline, among all children, boys and girls, by intervention group (bivariate analysis)

The average Hb concentration at baseline was 9.41 g/dL (SD = 1.84), with no statistically significant differences between intervention and control groups (Table 2). Mean Hb reached 10 and 10.1 g/dL at F1 and F2, again without any differences between groups. However, at endline Hb was statistically significantly higher (p = 0.04 in the intervention group; 10.62 g/dL (SD = 137)) compared to the control group (10.38 g/dL (SD: 1.40)). There were no statistically significant differences between intervention groups when data were disaggregated by gender (Table 2).

**Table 2:** Hemoglobin concentration and anemia prevalence among children 24 to 59 months of age at baseline in intervention group (MNFY+BCC) and control group (BCC).

	All			p	Boys			p	Girls			p
	All	Intervention	Control		All	Intervention	Control		All	Intervention	Control	
**Mean Hemoglobin, g/dL, N, mean (SD)**												
Baseline	449	204	245	0.10	231	102	129	0.06	218	102	116	0.71
	9.41 (1.84)	9.25 (1.90)	9.54 (1.78)		9.27 (1.78)	9.02 (1.96)	9.47 (1.60)		9.47 (1.83)	9.47 (1.83)	9.62 (1.97)	
F1	370	170	200	0.98		89	108	0.76		81	92	0.62
	10.00 (1.49)	10.00 (1.48)	10.00 (1.51)		9.86 (1.62)	9.86 (1.62)	9.77 (1.46)		10.21 (1.43)	10.15 (1.30)	10.26 (1.54)	
F2	376	171	205	0.26		83	104	0.46		88	101	0.30
	10.10 (1.45)	10.20 (1.33)	10.02 (1.54)		10.11 (1.42)	10.11 (1.42)	9.94 (1.44)		10.19 (1.47)	10.29 (1.24)	10.10 (1.65)	
Endline	423	191	232	0.04		95	122	0.23		96	110	0.18
	10.49 (1.39)	10.62 (1.37)	10.38 (1.40)		10.36 (1.44)	10.51 (1.44)	10.24 (1.44)		10.63 (1.33)	10.74 (1.31)	10.54 (1.35)	
**Anemia prevalence, Hb<11 g/dL, %**												
Baseline	0.80	0.79	0.80	0.80	0.83	0.83	0.82	0.82	0.77	0.75	0.78	0.73
F1	0.72	0.73	0.69	0.34	0.76	0.75	0.76	0.94	0.66	0.72	0.61	0.19
F2	0.71	0.67	0.75	0.17	0.73	0.66	0.78	0.08	0.70	0.68	0.71	0.67
Endline	0.61	0.59	0.62	0.39	0.63	0.61	0.65	0.68	0.58	0.57	0.60	0.57
**Severe anemia prevalence, Hb<7g/dL, %**												
Baseline	0.12	0.15	0.09	0.04	0.12	0.19	0.06	<0.01	0.12	0.12	0.12	0.87
F1	0.03	0.04	0.03	0.56	0.05	0.07	0.03	0.22	0.02	0.01	0.03	0.37
F2	0.03	0.01	0.04	0.11	0.03	0.02	0.04	0.58	0.02	0.00	0.04	0.10
Endline	0.01	0.01	0.02	0.56	0.02	0.02	0.02	0.81	0.01	0.00	0.02	NA

All p-values obtained using a mixed linear regression model testing the difference between study groups, controlling for clustering at the concession level (random effect).

Number of children (N) for hemoglobin concentration apply also for anemia and severe anemia at baseline, F1, F2 and endline. Values are means (SD) or percentages.

Hemoglobin in g/dL. Anemia defined as Hb<11g/dL. Severe anemia defined as Hb<7g/dL. Abbreviations: BCC: behavior change communication campaign; F1: follow-up survey 1; F2: follow-up survey 2; Hb: hemoglobin; MNFY: micronutrient fortified yoghurt

Up to 80% of all children were anemic at baseline. The prevalence of anemia was not statistically different in bivariate analysis, between intervention and control groups at any of the measurement points for all children as well as for boys and girls analyzed separately. Overall, the prevalence of anemia decreased by close to 20 pp in the study population from baseline to endline, with no statistically significant differences between the groups (it decreased from 79% to 59% in intervention group; and from 80 to 62% in control group).

The prevalence of severe anemia was 12% at baseline and was unbalanced between intervention and control group (15% in intervention group and 9% in control group, p = 0.04). Among boys, the prevalence of severe anemia was much higher in the intervention group, with 19% of boys severely anemic vs 6% in the control group at baseline, p<0.01. This imbalance was not seen with girls as 12% were severely anemic in both groups at baseline. The prevalence of severe anemia decreased drastically between baseline (12%), F1 (3%), F2 (3%) and endline (1%), in the sample as a whole, for both intervention groups and in both boys and girls.

### Referral for severe anemia at baseline

At baseline, 12% of children were severely anemic. Among them, only half (27/53) were taken for treatment and received at least one bottle of iron syrup at the health center, in spite of the fact that the project provided referral, and covered transport and treatment costs. The long distances and the fact that children diagnosed as severely anemic may have no evident clinical signs of illness may explain the low level of follow-up for referral to health posts. There was a difference in the proportion of children who were taken for treatment and received iron treatment at baseline between the two study groups; 32% (7/22) in the control group vs 64% (20/31) in the intervention group, p = 0.02. The proportion of boys and girls taken for treatment was 46% (12/26) and 56% (15/27) respectively (p = 0.51).

### Impact of the intervention on hemoglobin concentration and anemia at different time-points, for all children, boys and girls

The increase in Hb concentration was significantly greater in the intervention (MNFY+BCC) compared to the control (BCC) group for all children at each point in time: it was greater by 0.31 g/dL,95%CI (-0.02; 0.61), 0.42 g/dL, 95%CI (0.12;0.72) and 0.55 g/dL, 95%CI (0.27; 0.84) at F1, F2 and endline respectively (baseline and control group serve as reference for the interactions between intervention and F1, F2 and endline) (Table 3).

**Table 3:** Impact of MNFY on mean Hb concentration (g/dL) among children 24 to 59 months of age at different time-points, among all children, boys and girls (mixed models, random effect at cluster and individual level).

	All		Boys		Girls	
N	447	p	229	p	218	p
	Coeff (95%CI)		Coeff (95%CI)		Coeff (95%CI)	
Intervention	-0.18 (-0.48;0.12)	0.23	-0.36 (-0.77;0.05)	0.09	-0.07 (-0.49;0.34)	0.73
Time (ref = Baseline)						
F1	0.45 (0.24;0.65)	<0.001	0.34 (0.07;0.61)	0.01	0.57 (0.27;0.87)	<0.001
F2	0.51 (0.31;0.71)	<0.001	0.51 (0.24;0.79)	<0.001	0.50 (0.21;0.79)	<0.01
Endline	0.84 (0.65;1.03)	<0.001	0.78 (0.52;1.03)	<0.01	0.90 (0.62;1.18)	<0.001
Intervention x Time (ref = control & baseline)						
Intervention x F1	0.31 (0.02;0.61)	0.04	0.48 (0.08;0.89)	0.02	0.12 (-0.32;0.56)	0.59
Intervention x F2	0.42 (0.12;0.72)	<0.01	0.58 (0.16;0.99)	<0.01	0.27 (-0.15;0.69)	0.21
Intervention x Endline	0.55 (0.27;0.84)	<0.001	0.72 (0.34;1.12)	<0.001	0.38 (-0.03;0.80)	0.07
Child’s age at baseline	0.02 (0.01;0.03)	<0.001	0.03 (0.01;0.04)	<0.001	0.01 (0.00;0.03)	0.04
Boys	-0.24 (-0.46;-0.02)	0.03	-		-	
Iron treatment at baseline	-1.42 (-1.88;-0.96)	<0.001	-1.09 (-1.68;-0.51)	<0.001	-1.55 (-2.27;-0.83)	<0.001
Wealth Index	0.52 (-0.09;1.13)	0.09	0.54 (-0.25;1.33)	0.18	0.58 (-0.31;1.47)	0.20
Mother’s age	0.02 (0.01;0.04)	<0.01	0.02 (0.01;0.04)	<0.01	0.00 (-0.01;0.02)	0.71
Intercept	8.16 (7.46;8.86)	<0.001	7.32 (6.37;8.27)	<0.001	8.61 (7.64;9.58)	<0.001

Only adjusted models on covariates are presented (coefficient not varying in non-adjusted models). P-values obtained with mixed regression models, using random effect at child-level to take into account correlations between repeated measures (unstructured matrix of variance-covariance) and random effect at the concession-level (cluster), testing the interactions intervention*time. Values are means (95%CI). Abbreviations: F1: follow-up survey 1; F2: follow-up survey 2; Hb: hemoglobin; MNFY: micronutrient fortified yogurt

The impact of the intervention on Hb concentration was confirmed in boys, with Hb increasing by 0.48 g/dL, 95%CI (0.08; 0.89), 0.58 g/dL, 95%CI (0.16; 0.99) and 0.72 g/dL, 95%CI (0.34; 1.12) more in intervention compared to control group at F1, F2 and endline respectively (baseline serves as reference), but not in girls (none of the interaction terms between intervention and time are statistically significant). However, girls gained an average of 0.90 g/dL, 95%CI (0.62; 1.18) between baseline and endline, regardless of the intervention group (effect of time), more so than boys who gained an average of 0.78 g/dL, 95%CI (0.52; 1.03).

Although overall, anemia prevalence decreased by 19 pp for all children (Table 2), there was no impact of the intervention on anemia prevalence using repeated measures through the year (S3 Table) (interactions between intervention and time were not statistically significant). The data suggest that the lack of detectable impact is likely due to the fact that children in both intervention and control group experienced a similar (and large) reduction in anemia over the 1 year study period (20 pp in intervention and 18 pp in control group).

Additional analyses also suggest that the lack of impact of the intervention on anemia, in spite of a significant impacts on Hb, may be due to the fact that Hb levels remained low (below the 11 g/dL cut-off point that defines anemia) at each measurement. When using an arbitrary lower cut-off point to define anemia (10 g/dL), for example, we found a marginally significant impact of the intervention on anemia prevalence after one year of intervention (a decline in anemia prevalence between baseline and endline, that was 9 pp greater in intervention compared to control group (95%CI (-0.19, 0.01)).

### Maternal knowledge on anemia prevention

Maternal knowledge regarding the health consequences of anemia and the dietary sources of iron was very low at baseline—only 8% of mothers recalled more than two out of a list of five health consequences of anemia for children (i.e. difficulty at school, impaired development, slow growth, weak immunity defenses, fatigue) and the same percentage could list two out of three iron-rich foods (i.e. liver, fish or meat), with no difference between intervention groups. Knowledge improved on both topics over the study period—with 19% of mothers correctly identifying two out of five health consequences of anemia (p<0.001) and 21% being able to list two out of three iron-rich foods at endline (p<0.001). As expected, given that the BCC intervention was offered to both intervention and control groups, there were no statistically significant differences in knowledge improvement over the study period.

## Discussion

This study is the first to document the impact of a nutrition-sensitive value chain intervention on child nutrition outcomes, using a cluster-randomized control trial. The intervention used the logistics of a supply chain for milk collection to deliver a MNFY produced with the milk collected by pastoralists. The dairy value chain intervention was successful in improving Hb among nutritionally vulnerable preschool children living in a resource-poor, remote area of Senegal. The MNFY also served as an effective incentive for farmers to increase regularity of milk deliveries when used as part of a contractual relation with the dairy factory.

The MNFY had a positive impact on Hb in children 24–59 months of age at baseline, after one year of intervention. Mean Hb increased by 0.55g/dL, 95%CI (0.27; 0.84) more in the intervention compared to the control group, and this impact was statistically significant in the overall sample, and in boys (0.72 g/dL, 95%CI (0.34; 1.12)), but not in girls.

The overall magnitude of impact on Hb found in our study is in line with a Cochrane review of randomized trials using micronutrient sprinkles (MNPs) distributed for durations ranging from 2 to 12 months, which documents a mean impact of 0.59 g/dL, 95%CI (0.32; 0.85) for children receiving MNPs compared to children receiving no treatment or placebo [13]. A 4-month study assessing the efficacy of maize porridge enriched with MNP (with 2.5 mg iron as NaFeEDTA, a level of iron fortification similar to the one used in our study) in children 12–59 months of age in Kenya, found that Hb increased by approximately 0.27 g/dL, 95% CI (0.04; 0.51) compared to children from the control group [14]. A meta-analysis pooling data from 54 randomized control trials found a significant mean increase in Hb of 0.42 g/dL, 95% CI (0.28; 0.56) in groups receiving fortified or biofortified foods compared to control groups [15]. According to this meta-analysis, the impact was obtained in settings free of infections such as malaria, when iron-EDTA was used as fortificant, and where initial concentrations of Hb were low, allowing more room for improvement. Our study meets these criteria, as the Richard Toll area has nearly achieved malaria eradication [16], the iron used to fortify MNFY was a highly absorbable iron-EDTA, and the Hb concentration at baseline was very low, particularly among boys.

The MNFY did not lead to statistically significant differential decreases in anemia prevalence between intervention and control groups, in spite of the significant improvements found in Hb. There are several potential explanations for this finding. First, given the low initial Hb levels at baseline (mean = 9.41 in whole sample; 9.27 in boys), the average improvement of 1 g/dL over the study period would be insufficient to allow children at the low end of the distribution to reach the cut-off point of 11 g/dL that defines anemia. This hypothesis is supported by our analyses showing that using a lower (arbitrary) cut-off point of 10 g/d to define anemia resulted in a marginally significant difference in the reduction of anemia between intervention and control (9 pp; p = 0.07).

The next question is why were there improvements in Hb and reductions in anemia in both intervention and control groups? One plausible explanation is the fact that the BCC intervention was provided to both intervention and control groups and that mothers’ knowledge about anemia and iron-rich foods improved (albeit moderately) in both groups. In addition, better environmental conditions coming from better rainfalls in 2014 compared to 2013 could explain overall better nutritional status among the children in our cohort. Another potential explanation could be a decrease in malaria and schistosomiasis infections responsible for anemia, or deworming campaigns happening in the area during the study period. In 2012, the incidence of malaria attacks was very low, a finding attributed to high vector control coverage for malaria in Richard Toll and a low human density area [16]. We are unaware of additional changes between 2012 and 2014 (before and during our study). We also have no information on changes in rates of schistosomiasis transmission in the region or deworming campaigns that could have affected anemia prevalence in preschool children during the course of the study. Lastly, although we found no evidence of widespread consumption of MNFY in the control group, we cannot rule out the potential of spill-over effects given that may concessions (our unit of randomization) in intervention and control groups were in close proximity, and the MNFY was delivered at the same milk collection points used for farmers from both intervention and control groups.

Our study has several strengths. First, the iron fortificant used, iron-EDTA, has proven bioavailability in phytate-rich meals like millet, with absorption levels 2 to 3 times higher than with other types of iron, while also increasing the absorption of iron present in other foods included in the diet [17]. It has been shown to be safe for everyday use, even at a higher dosage and at large scale [18]. The calcium present in yoghurt could reduce the bioavailability of iron, but the amount provided in one sachet of MNFY was 50 mg, much lower than the level (300 mg) estimated to cause maximal inhibitory effect on iron [19]. Second, the study used repeated measurement on a cohort of children, retention in the study was high, and the statistical method used to assess impact (mixed regression model, which takes into account repeated measures in the same individual) was well suited for our data. Third, the intervention targeted women by delivering the MNFY product to collection points easily accessible to them. A companion study shows that the intervention led to increases in women’s decisionmaking [8]. Empowering women has been shown to be a sustainable strategy for improving children’s health, even after the intervention ends [7].

The main limitation of our study is the reliance on Hb as our main indicator. Field logistics and financial aspects prevented us from using more specific biomarkers of iron deficiency and include measures of infections. Another limitation is the lack of information on the exact micronutrient composition of the 80 g sachet of MNFY (which contains 75% yoghurt, 25% of millet grains and iron, iodine, vitamin A and zinc fortificants). Finally, careful measurement of dietary intake and of the consumption of MNFY would have been useful to better understand sharing patterns and the contribution of the supplement to overall nutrient intake, as well as for use as a covariate in impact models, but such measures were beyond the scope of the study.

Several lessons emerged from this pilot study aimed at testing the impacts of a nutrition-sensitive value chain on child Hb and anemia status, using a rigorous randomized control trial. First, the nutritional incentive had a positive impact on milk production and delivery during the dry season, the impact on milk production of the intervention being larger in households where women controlled the milk contract, as they had more decision-making power over the milk production [20]. This result highlighted the importance of empowering women in projects aimed at improving children nutritional status like value chains interventions. Second, the value chain approach increased access to an affordable product (lower cost than MNPs) and reached a remote semi-nomadic population with little access to health services or nutrient-rich foods. Finally, the successful design and implementation of this project was made possible by the strong partnership between the private sector, an NGO, and a governmental nutrition agency right from the onset of the project.

The nutrition-sensitive dairy value chain approach used in this population with high anemia rates (and limited access to health services and iron-rich foods) showed to be an effective way to improve Hb in pre-school children. This study is the first to document, using a cluster randomized control trial, that a traditional dairy value chain project can be leveraged to improve nutrition in a remote pastoralist population of Northern Senegal. Innovative approach like value chains coupled with rigorous evaluations are needed to tackle children’s malnutrition and in particular micronutrient deficiencies in developing countries [7][21].

## Supporting information

S1 Consort Checklist (DOCX)

S1 Protocol Original study protocol. (DOCX)

S1 Table Household level of exposure (children in the household consuming MNFY during the 3 days prior to the survey) and children living in these HH, in the intervention group, all children, boys and girls, 24 to 59 months of age. (DOCX)

S2 Table Children surveyed by intervention group at different time points, all children 24 to 59 months of age, boys and girls. (DOCX)

S3 Table Impact of MNFY on prevalence of anemia (percentage points) among children 24 to 59 months of age with 4 measures, among all children, boys and girls (mixed models, random effect at cluster and individual level). (DOCX)
