# Supplementary material for: Delivery of iron-fortified yoghurt, through a dairy value chain program, increases hemoglobin concentration among children 24 to 59 months old in Northern Senegal: A cluster-randomized control trial
Source: PLoS One. 2017 Feb 28;12(2):e0172198. doi: 10.1371/journal.pone.0172198 (PMC5330480; doi:10.1371/journal.pone.0172198)
Supplement: S1 Protocol — (DOCX) [file pone.0172198.s002.docx]

Improving nutrition through agricultural contracts / Improving agricultural contracts through incentive linked to nutrition

Consumption of fortified Thiakry and effect on anemia amongst children ages 2 to 5 years in the zone of Richard Toll, Senegal.

**Cluster randomized trial to test the distribution amongst herders of fortified Thiakry as an incentive to increase milk collection during the dry season and decrease anemia amongst children ages 2 to 5 years**

**Principal Investigators**

Tanguy Bernard

Maximo Torero

Marie Ruel

Rahul Rawat

**Co-Investigators/Coordinators**

Agnes Le Port

Melissa Hidrobo

Samba Mbaye

**Research assistants**

Ligane Sene

Cheikh Ibrahima Diop

***Principal Investigators***

Tanguy Bernard

Market, Trade and Institution Division (MTID)

International Food Policy Research Institute (IFPRI)

Lot n°2 - Titre 3396-BP 24 063

Almadies, Dakar, Senegal

[t.bernard@cgiar.org](mailto:t.bernard@cgiar.org)

Maximo Torero

Market, Trade and Institution Division (MTID)

International Food Policy Research Institute (IFPRI)

2033 K St, NW

Washington, DC 20006-1002

USA

Marie Ruel

Poverty, Health and Nutrition Division (PHND)

International Food Policy Research Institute (IFPRI)

2033 K St, NW

Washington, DC 20006-1002

USA

[m.ruel@cgiar.org](mailto:m.ruel@cgiar.org)

Rahul Rawat

Poverty, Health and Nutrition Division (PHND)

International Food Policy Research Institute (IFPRI)

2033 K St, NW

Washington, DC 20006-1002

USA

[r.rawat@cgiar.org](mailto:r.rawat@cgiar.org)

***Co-Investigators/Coordinators***

Agnes Le Port

Poverty, Health and Nutrition Division (PHND)

International Food Policy Research Institute (IFPRI)

Lot n°2 - Titre 3396-BP 24 063

Almadies, Dakar, Senegal

[a.leport@cgiar.org](mailto:a.leport@cgiar.org)

Melissa Hidrobo

Poverty, Health and Nutrition Division (PHND)

International Food Policy Research Institute (IFPRI)

2033 K St, NW

Washington, DC 20006-1002

USA

[m.hidrobo@cgiar.org](mailto:m.hidrobo@cgiar.org)

Samba Mbaye

Universite Gaston Berger

Saint Louis

[sambambayeptci@yahoo.fr](mailto:sambambayeptci@yahoo.fr)

***Research assistants***

Ligane Sene

West and Central Africa Office

International Food Policy Research Institute (IFPRI)

Lot n°2 - Titre 3396-BP 24 063

Almadies, Dakar, Senegal

[L.Sene@cgiar.org](mailto:L.Sene@cgiar.org)

Cheikh Ibrahima Diop

Universite Gaston Berger

Saint Louis

[mamecheikhdiop@gmail.com](mailto:mamecheikhdiop@gmail.com)

***Partner institutions***

International Food Policy Research Institute (IFPRI)

Laiterie du Berger (LDB)

- Bagore Bathily/Isabelle Sultan/Arona Diaw

GRET

- Guillaume Bastard

Cellule de Lutte Contre la Malnutrition (CLM)

- Ndeye Khady Toure for CLM Dakar
- Aminata Ndoye for CLM Saint Louis

**SUMMARY**

**Justification of the study**

According to recent estimates, nearly one billion people still suffer from hunger. Malnutrition remains a major cause of morbidity and maternal and infant mortality in sub-Saharan Africa (FAO, 2010). Malnutrition includes chronic and acute forms, as well as deficits in essential micronutrients. Access to a rich and good quality diet remains difficult for many people. Poor households continue to have a diet low in diversity and poor in foods with essential micronutrients such as vitamin A, iron and zinc (Arimond, 2010). Iron deficiency in its most severe form leads to anemia. In Senegal, nearly 83% of children 12-23 months olds are anemic and 64.5% of children of the same age have an iron deficiency (MI, 2012).

In 2006, the Laiterie du Berger (LDB) began collecting milk from herding families living in the area of Richard Toll, in the Senegal River Valley. The originality of LDB is that it produces yogurt by collecting fresh milk twice a day in households situated within a 50 km radius around the factory. Over the years, LDB has developed a network of 700 suppliers on 4 main collection routes. However, during the dry season, LDB is faced with a significant drop in volumes and collections face great irregularity. One of the hypotheses brought forward to explain this phenomenon is that those who collect the milk (women, children, herders) are not always those who receive the payments. Another possibility is the sale of milk at markets in the dry season can fetch a higher price than that fixed by LDB.

This study aims to test a type of incentive among farmers so that they might provide milk to LDB more regularly during the dry season. The incentive will be distributed as a bonus, after agreeing upon a contract, with a target volume (corresponding to 1 liter of milk per lactating cow per day) and regularity in the frequency of milk collection, at the beginning of the dry season. The incentive tested will be a bonus dairy product produced by LDB itself: fortified Thiakry. This form of incentive corresponds to the "Value Chain" concept: the initial product is the milk produced by herders and the processing of the milk produces yogurt, to which millet and fortification are added. This results in a final product (fortified Thiakry) that will be returned to herders and has a value added compared to the initial product.

**Methodology**

**1. Overall objective, specific objectives, type of study, and number of subjects**

*Overall objective:* to demonstrate the "Value Chain" concept in a contractual agreement linking the Laiterie du Berger and milk producers in the area of Richard Toll, Senegal.

*Specific objectives:* to compare the results of a fortified Thiakry intervention versus a control group, concerning:
- Increasing the volume of milk and the frequency of collection during the dry season
- Reducing the prevalence of anemia among children aged 2 to 5 years

*Type of study:* cluster randomized control trial, comparing households receiving fortified (with iron) Thiakry to control households receiving no intervention. Children 2 to 5 years at baseline will be visited 6 months and 1 year from the beginning of the study.

*Number of required subjects:* 700 families of herders in total.

**2. Criteria for assessment**

*Main outcome of the intervention:* proportion of herders who have reached the objective defined at the beginning of study in the contract with LDB, in terms of volume and collection frequency for milk over the duration of the study

*Main outcome for the "Nutrition" component of the intervention*: proportion of children 2 to 5 years presenting severe, moderate, and mild anemia (defined thresholds respectively are <7, between 7 and 9.9, and between 10 and 10.9 g/dl of hemoglobin).

*Secondary outcomes of the "Nutrition" component (at 6 and 12 months of follow-up):*
- Mean level of hemoglobin (in g/dl)
- Proportion of children 2 to 5 suffering from stunting (Height-for-Age Indices <-2SD)
- Proportion of children 2 to 5 years suffering from acute malnutrition (Weight-for-Height Indices <-2 SD)
- Proportion of children 2 to 5 years who are underweight (Weight-for-Age Indices <-2 SD)
- 24 hour food consumption diversity (qualitative)

- Household food consumption over 7 days (consumption frequency of different food groups)

**3. Selection Criteria**

All households of herders supplying milk to the Laiterie du Berger and having children 2 to 5 years in the household, who agree to participate in the study

**4. Trial Procedures**

- An information-sharing meeting will be held on each collection route, gathering all the heads of households to: explain the terms of the study and the targets to achieve in terms of milk collection; perform the intervention lottery to define the treatment and control groups; distribute the explanatory note of the study and the information sheet on anemia (in French and Fulani); and distribute a form indicating willingness to participate in the study (to be brought, signed, to a second meeting 10 days later)
- An awareness-raising campaign will be implemented in the study zone by the Cellule de Lutte contre la Malnutrition (Unit for the Fight against Malnutrition; CLM) in January 2013, through community representatives of ASBEF in villages and through radio commercials, in order to inform mothers on good feeding practices and explain the problems of anemia. This campaign will take place in the study zone for 6 months.
- A first household survey will take place in January 2013, to gather baseline data through the form of a household questionnaire (household composition, herding/cow data, socio-economic data, household consumption etc.); anthropometric data; and hemoglobin measures of children 6 months to 5 years old and their mothers, using HemoCue®.

- A distribution phase of incentives (fortified Thiakry) for 6 months, from January 2013 to July 2013.
- A second phase of data collection in July 2013, through household questionnaire and measurement of hemoglobin levels as during the baseline data collection.
- A third phase of data collection in January 2014, through household questionnaire and measurement of hemoglobin levels.

**5. Location and duration**

The study will take place in the department of Dagana, in the area of ​​Richard Toll, amongst farmers of the area supplying milk to the Laiterie du Berger. The study area covers the zones of the Walo and the Diery. The implementation of the "herding" arm of the study (identifying contract objectives for farmers, information meetings etc.) will be entrusted to GRET. The intervention will be coupled with an awareness campaign in the area (the “nutrition” arm). This will cover good dietary practices for children, as well as anemia and means of prevention. It will be led by CLM and its executing partner ASBEF, through their networks of health workers, who are in close contact with the population. The intervention will last 6 months and an evaluation will be conducted at 6 months and 12 months.

**SUMMARY-TABLE OF CONTENTS**

1. Justification of the study
2. Objectives of the study
3. Criteria for assessment
4. Location of the study
5. Design of the study
6. Characteristics of subjects
7. Number of subjects
8. Conditions of the lottery
9. Fortified Thiakry
10. Test Procedures
11. Biometrics and data analysis
12. Administrative procedures and considerations/Practical aspects
13. Publications
14. Financing
15. Bibliography
16. Annexes

# 1. Justification of the study

Current estimates suggest that there are almost 1 billion hungry people in the world (FAO 2010). In sub-Saharan Africa, a substantial share of death and disease results from high levels of undernutrition among mothers and children. Indeed, maternal and child undernutrition has long-lasting, irreversible effects on the development, health, and economic productivity of individuals, communities, and nations. Globally, maternal and child undernutrition is the underlying cause of 3.5 million deaths, 35% of the disease burden in children younger than 5 years, and 11% of total global disability adjusted life years (DALYs) (Black et al. 2009) . Reducing undernutrition is a key factor underpinning the achievement several Millennium Development Goals (MDGs).

Undernutrition encompasses both chronic and acute forms, as well as deficiencies of essential micronutrients. Access to nutrient-rich foods and to diets of adequate quality remains a major impediment towards progress in tackling the problem of undernutrition. Typically, poor households subsist on low-diversity, staple-based diets and lack access to nutritious foods that are rich in essential micronutrients such as vitamin A, iron, and zinc (Ruel et al. 2003; Arimond et al. 2010). Inadequate intake of these micronutrients have far reaching health, nutrition, and developmental consequences both in the short and the long term (Black et al. 2008; MI, 2009). Women of reproductive age and infants and young children are particularly vulnerable to the consequences of micronutrient deficiencies because their life stages correspond with high micronutrient intake requirements (Black et al. 2008; MI, 2009).

A deficit in iron, in its most severe form, leads to iron-deficiency anemia. Due to the facility of measuring hemoglobin levels, the prevalence of anemia has often been used as a proxy for measuring iron-deficiency anemia. This approach is sufficient in areas where iron deficiency is known to be the major cause of anemia. This is not the case in areas where the cause of anemia is much more complex. While it has been estimated that at least 50% of cases of anemia can be attributed to iron deficits (Stoltzfus, 2003), these figures vary considerably depending on the geographic area and age groups concerned. In its latest report on anemia in the world in 2009, the WHO indicates an anemic prevalence among children 0-5 years of 68% in Africa (McLean, 2009). A recent study carried out in Ivory Coast has shown that 40-50% of children and women were anemic and that iron-deficiency anemia was associated with 50% of cases of anemia among school-age children and women, while this figure reached 80% among children of pre-school age (2-5 years) (Staubli, 2001).

High prevalence of anemia in a population is also a reflection of low levels of health. There are many causes of anemia in developing countries. Among them, in addition to iron deficiency, other micronutrients such as folic acid, vitamin B12, and vitamin A appear to be involved. Infectious diseases such as malaria, HIV, and intestinal helminth infections (hookworm and schistosomiasis from Schistosoma haematobium) (Stoltzfus, 2000), as well as hemoglobinopathies (sickle cell disease, thalassemia) (Tolentino, 2007), also play an important role.

The consequences of anemia and iron deficiency on the health of the individual are multiple (Berger, 2002). In adults, they result in a decrease in physical capacity (Gardner, 1977) and productivity (Basta, 1979). In pregnant women, the impact on mortality that is attributable to anemia has been calculated respectively at 6.37, 7.26, and 3.0% respectively for Africa, Asia and Latin America (Brabin, 2001). Anemia and iron deficiency increase the risk of morbidity and fetal and neonatal mortality, of prematurity, and of low infant birthweight (Viteri 1994). In children, iron deficits may cause cognitive dysfunction and affect their development potential (Walker, 2007; Eden, 2005).

Different targeted interventions to provide iron supplements to vulnerable populations have been evaluated (Berger, 2002). Approaches based on increased consumption of iron and food diversity are important strategies for the prevention of iron deficiency and iron-deficiency anemia in the general population. However, integrated approaches that combine supplementation interventions in iron and other health measures (such as deworming, actions to combat malaria, mass treatment for schistosomiasis) were necessary in areas where iron deficiency was not the only cause of anemia. Education and respect for good dietary practices are also essential (Buttha, 2008). Finally, the implementation of interventions in the fight against micronutrient deficiencies (through supplementation, fortification, and dietary modification) should be integrated in agriculture, welfare, and/or health programs (Olney, 2012).

Senegal has seen significant progress in combating malnutrition over the past two decades. Alleviating child malnutrition has been high on the Senegalese policy agenda, with a dedicated unit – the Cellule contre La Malnutrition (CLM) – coordinating major activities in-country and reporting directly to the prime ministers’ office. According to the 2011 DHS report, 83% of children under 5 years and 54% of mothers are anemic in Senegal. According to the same report, growth delays (indicator of chronic malnutrition) affect 27% of children, with thinness or wasting (acute malnutrition indicator) affecting 10% of children and 18% being underweight (DHS 2011).

According to a 2012 report of CLM on the iron status of children aged 12-59 months across Senegal, the prevalence of anemia is 83% for children 12-23 months and declines to 49% for children 48-59 months (MI, 2012). Analyses on iron status indicate that 64.5% of children have an iron deficiency, ranging from 64.3% for children 12-24 months and decreasing to 42.5% for children 48-59 months. Finally, according to the latest Smart 2012 survey, conducted by CLM in the department of Dagana among children 6-59 months, acute malnutrition affected 11.4% of children (as compared to 8.80% nationally), stunting impacted 13.6% (compared to 15.50% nationally), and 15.5% were underweight (compared to 14.50% nationally) (CLM, 2012).

In 2006, the Laiterie du Berger (LDB), a social venture later supported by Danone Communities, Credit Agricole, the Grameen Foundation, and other partners, started to collect milk from pastoralist households living near the town of Richard Toll in the Senegal River Valley. LDB’s business model is to produce high-quality products (primarily yogurt) for mostly urban populations, using fresh milk that it collects itself twice a day from households living within 50 km of its processing plant. Over the years, LDB has built up a reliable base of 700 milk suppliers on 4 main collection routes. At the end of the month, suppliers come to LDB and collect the payment for their month’s production. Part of these payments can be used to purchase discounted animal feeds, which appears to be an important incentive for suppliers. They may also receive an in-kind advance (in animal feeds) of up to 30% of the past month’s production, if they face specific liquidity and/or animal feed needs.

The current study aims to test a type of incentive among farmers to encourage them to provide milk to LDB more regularly during the dry season, while investigating and explaining the mechanisms involved. The incentive will be distributed as a bonus, after a contract with a target volume (corresponding to 1 liter of milk per lactating cow per day) and regularity in the frequency of milk collection has been agreed upon at the beginning of the dry season. The incentive tested will be a dairy product produced by LDB itself: fortified Thiakry. This form of incentive corresponds to the "Value Chain" concept^^[[1]](#footnote-1)^^: the initial product is the milk produced by herders and the processing of the milk produces in yogurt to which millet and fortification are added. This results in a final value-added product (fortified Thiakry) that will be made accessible to herders.

# 2. Objectives of the study

*Overall objective:* to demonstrate the "Value Chain" concept in a contractual agreement linking the Laiterie du Berger and milk producers in the area of Richard Toll, Senegal.

*Specific objectives:* to compare the results of a micronutrient-fortified Thiakry intervention with a control group, specifically concerning:
- Increasing the volume of milk and the frequency of collection during the dry season
- Reducing the prevalence of anemia among children aged 2 to 5 years

3. Criteria for assessment

*Main outcome of the intervention:* proportion of herders who have reached their contract objective defined in the beginning of study, in terms of volume and collection frequency for milk over the duration of the study

*Main outcome for the "Nutrition" component:* proportion of children 2 to 5 years presentingsevere, moderate, and mild anemia (defined thresholds respectively are <7, between 7 and 9.9, and between 10 and 10.9 g/dl of hemoglobin).

*Secondary outcome of the "Nutrition" component (at 6 and 12 months of follow-up):*- Mean level of hemoglobin (in g/dl)
- Proportion of children 2 to 5 suffering from stunting (Height-for-Age Indices <-2SD)
- Proportion of children 2 to 5 years suffering from acute malnutrition (Weight-for-Height Indices <-2 SD)
- Proportion of children 2 to 5 years who are underweight (Weight-for-Age Indices <-2 SD)

- Household food consumption

- Children’s dietary diversity

*Definitions:*

- Mild anemia in children: level of hemoglobin between 10 and 10.9 g/dl of blood

- Moderate anemia in children: level of hemoglobin between 7 and 9.9 g/dl of blood

- Severe anemia in children: level of hemoglobin less than 7 g/dl of blood

- Stunting (Height-for-Age Indices <-2SD)

- Acute malnutrition or wasting (Weight-for-Height Indices <-2 SD)

- Underweight (Weight-for-Age Indices <-2 SD)

# 4. Location of the study

LDB is located in the area of Richard Toll, along the Senegal River Delta in the administrative department of Dagana. Collection routes are located in the Dieri and in the Walo (flood zone near the river, where rice is commonly grown). The herding in the Walo is of a more sedentary/agro-pastoral nature whereas the herding in the Dieri is generally pastoral with transhumance (Parisse, 2011).


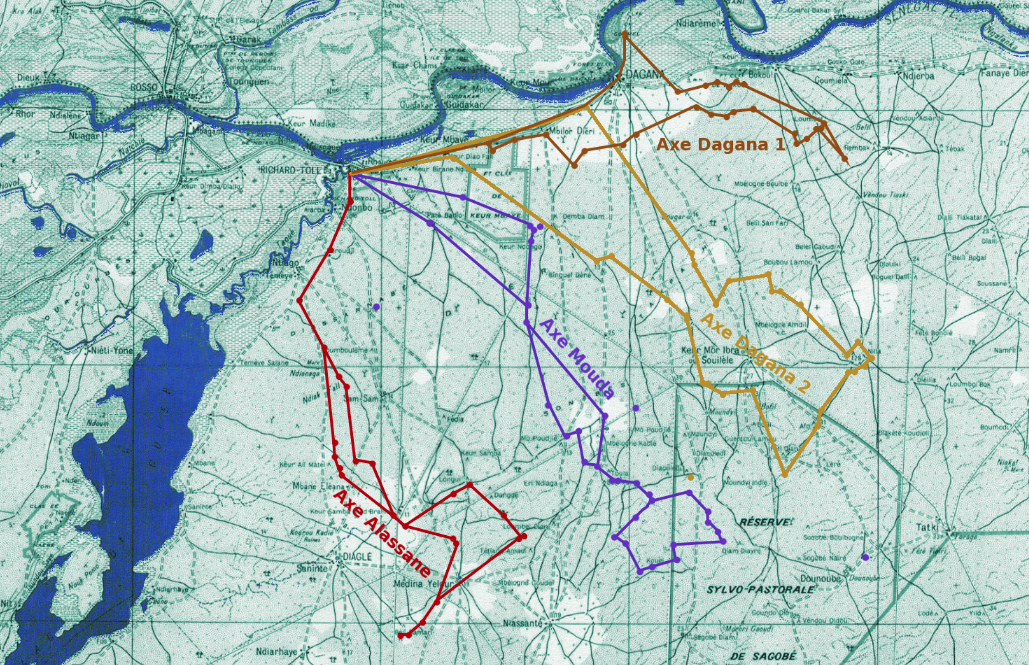


*Figure 1: Collection routes around Richard Toll, Senegal*

# 5. Study design

The study will be randomized and single-blind. Of the 4 collection routes (Figure 1), 500 milk containers are collected every day, corresponding to an average of 2 households of herders per container. Randomization will be made at the level of household concessions, with certain concessions receiving fortified Thiakry and others making up the control group.

# 6. Subject characteristics

# *Selection Criteria:*

- All households of herders supplying milk to the Laiterie du Berger and having children 2 to 5 years in the household, who agree to regularly provide milk to LDB

- Signature of an agreement to participate in the study by the head of household and his/her spouse(s) and signature of the informed consent by the mother of the children

*Non-inclusion criteria:*

- Refusal to participate in the study

*Exclusion criteria:*

- Subjects may discontinue participation in the study at any moment

# 7. Number of subjects

Calculating the number of required subjects is based on the main assessment criterion of the nutrition component of the study: anemia. Considering that the consumption of fortified Thiakry by children has the potential to reduce the prevalence of anemia by 15%, from an average prevalence of 75% to 60%, the number of required subjects is 165 subjects per group, totally 330 subjects, with 80% power and 5% alpha (unilateral test). This is a cluster trial, with the the concession as the randomization unit. We have therefore taken into account an intracluster correlation coefficient of 0.3, which gives an estimate of 430 children aged 2-5 years required. Estimating an attrition of 20%, we would need to recruit 550 children. Moreover, it is likely that not all households will participate in the study (largely due to the phenomenon of transhumance in the area) and that not all households will achieve their objective in terms of milk production in order to obtain the incentive. Among children in the group receiving the fortified Thiakry, there is no guarantee that all of the product will be consumed and at the necessary frequency (every day) to see a reduction of anemia. We therefore estimate that another 20% of the number of households may ineligible for analysis, bringing the total number of subjects required to 660 children. As the number of households of the area is approximately 700, with an average of 2 children in the age group of interest per household, we are confident that we will be able to access the necessary number of children.

# 8. Conditions of the lottery

The two groups will be chosen by lottery at the first information-sharing meeting. The draw will define the households receiving free fortified Thiakry for all children aged 2 to 5 years within their household (treatment group) and the households not receiving fortified Thiakry during the study (control group), who will receive in kind compensation at the end of the study as thanks for their participation.

# 9. Fortified Thiakry

- 1. **Premix Composition used by LDB for the Thiakry**

The premix for Thiakry fortification is developed by Danone R&D laboratory in France, for Danone Communities, which is a stockholder of LDB. The table of composition has been emitted from this document, due to privacy of information. It is shared in % of Recommended Dietary Allowance (RDA) for different targets (children from 1 to 3 years old, children over 3 years, and pregnant women) in Annex 1.

- 1. **Mechanism of action**

Use of iron EDTA (NaFeEDTA) for food fortification is becoming increasingly common, due to its higher absorption potential compared with sulfate iron, which was previously more common. Its absorption is 2 to 3 times greater than iron sulfate or iron fumarate (FAO/WHO, 2006) and it also increases the rate of absorption of naturally occurring iron (Van Stuijvenberg, 2008). EDTA iron is absorbed by normal physiological mechanisms. When NaFeEDTA is present in a meal, half of the iron is exchanged with naturally occurring iron and EDTA partially protects iron from inhibitory effects. Cereal inhibitors of iron absorption are phytates (i.e. sorgho) and polyphenols (i.e. millet) (Lestiennes, 2004). EDTA iron is particularly effective when added to cereals rich in absorption inhibitors (Troesch, 2010; De-Regil, 2011).

Iron bioavailability depends on dietary characteristics of population (WHO/FAO, 2006). According to guidelines on food fortification with micronutrients (Allen, 2006), there are 3 levels of bioavailability used to characterize typical diets.

- Low 1–9%: Simple, low-diversity diet based on cereals, roots or tubers, with negligible amounts of meat, fish, poultry or ascorbic acid-rich foods. Diet high in foods that inhibit iron absorption such as maize, beans, whole wheat flour, and sorghum.

- Intermediate 10–15%: Diet of cereals, roots or tubers, with some foods of animal origin (meat, fish or poultry) and/or containing some ascorbic acid (from fruits and vegetables)

- High >15%: Diversified diet containing greater amounts of meat, fish, poultry and/or foods high in ascorbic acid.

According to a 2012 study performed on micronutrient deficiencies in Senegal, daily meat consumption is rare in children at a national level (MI/COSFAM, 2012). 40.2% eat beef one to 3 times a week and 43.6% once a month. Globally, Senegalese children are not high consumers of leafy vegetables. Main leaves consumed are hibiscus, moringa, and baobab: 46.4 % consume hibiscus leaves at least one to 3 times a week and 45.6% rarely or never; 36.2% consume moringa leaves at least one to 3 times a week and more than the half (52.1%) rarely or never; and 34.5% consume baobab leaves at least one to 3 times a week and 53.9 rarely or never. Oranges (44.7%) and lemons (33.1%) are regularly consumed by children (at least once a week). On average, more than half of children occasionally or rarely eat fruits rich in vitamin C.

In the north of Senegal, the typical diet is rich in cereals (millet, rice) but poor in fish, poultry, and vitamin C-rich fruits. Meat consumption may be higher than the rest of Senegal but likely insufficient to reach a level of bioavailability higher than 10%. The level of green leafy vegetables, oranges, and lemons is likely inferior to the rest of the country. We therefore estimate that the level of iron bioavailability is between 5 and 10%.

With these low levels of iron bioavailability in typical diets (estimated between 5 and 10%), the dose of iron recommended by FAO and WHO is between 5.8 mg/day and 11.6 mg/day for children 1-3 years old and between 6.3 mg/day and 12.6 mg/day for children 4-6 years-old (WHO/FAO, 2006). However, using EDTA iron, absorption rates are increased by 2 or 3 times. Doses to cover daily needs must be divided according to the same rates.

Iron fortification levels used in another study performed in Bengladesh in infants 6-24 months was 3.3 mg of fumarate iron, which allowed the observation of a significant decrease of proportions of anemic children (Talukder, 2009). A study performed in Kenya in school children showed significant results with lower doses of EDTA iron (2.5 mg) in micronutrients powder (MNP) spread on a maize porridge for household-level fortification (Macharia-Mutie, 2012). In Kenya, another study showed that MNP containing 2.5 mg of iron EDTA and rich in vitamin C and phytases gave the same results (Troesh, 2011).

- 1. **Toxicity-Safety**

EDTA is a food additive. Its use has been approved by the FAO/WHO Expert Committee on Food Additives at the dose of 0.2 mg of iron per kg of bodyweight and per day (FAO/WHO, 2007). In to a recent study, a dose of 2 mg of NaFeEDTA was administered every day to children between the ages of 6 to 8 months, without exceeding recommended daily doses. EDTA iron is not toxic, according to different simulated scenarios corresponding to variable prevalence of underweight in children (thus likely able to surpass accepted limits per kg of weight) (Yang, 2011). In a study performed in China, a millet porridge was fortified with 2 mg of iron FeSO₄ or NaFeEDTA (first study) and with 4 mg of FeSO₄ iron or a mix of 2 mg of FeSO₄ and NaFeEDTA (second study) and given to children aged 24 to 31 months to test absorption of different combinations in a complementary product rich in phytates, with no adverse effects being reported (Chang, 2012).

# 10. Test Procedures

**10.1.** **Information and study recruitment**

A meeting will be held along each milk collection route, gathering all heads of households, in order to explain:
- Causes and consequences of anemia and the fact that LDB will make available to herders fortified Thiakry that fights against anemia
- The terms of the study, which are:

- the objectives in terms of milk collection
- the lottery defining the two intervention groups: the households receiving free fortified Thiakry for all children aged 2 to 5 years in the household and the households receiving no incentive but having the opportunity to buy fortified Thiakry directly from LDB if they wish
- the number of data collection rounds by enumerators for households etc.

- That mothers and children will have a drop of blood collected (finger prick) to measure their hemoglobin levels. The results will be immediately available and provided to families. In cases of severe anemia in children, IFPRI will facilitate transportation and will cover the price of the consultation and the cost of treatment at the nearest health center.

Household heads will leave the information meeting with an explanatory note of the study (in French and Fulani) and a form of intent to participate in the study (in French and Fulani). The form must be returned, signed by the head of household and their spouse, at the second meeting 10 days later, which will be establish the milk collection targets (contracts with LDB).

When the enumerators go to the households to begin data collection, they will read a document covering all the important aspects of the study and will obtain signature on a two-part informed consent form, targeting the household heads and the mothers.

**10.2.** **Awareness-raising campaign**

An awareness campaign will be implemented in the area by the Cellule de Lutte contre la Malnutrition (Unit for the Fight against Malnutrition; CLM) in December 2012, through community representatives of the Association Sénégalaise pour le Bien-Etre Familial (Senegalese Association for Family Well-Being; ASBEF), to inform mothers on good feeding practices and explain issues related to anemia. The awareness campaign will be conducted throughout the duration of the study through discussion groups at markets, health centers, and gathering areas, and through radio advertisements.

**10.3.** **Malaria, schistosomiasis, helminths**

As anemia often has multifactorial causes, the WHO recommends an integrated approach to fight against anemia (WHO/UNICEF, 2004). To assess the impact of distributing fortified Thiakry on anemia in the study area, it is necessary to take into account the risk of infection through malaria and helminth infections such as schistosomiasis, which may vary from one zone to the other (Staubli, 2001).

Malaria in Senegal is seasonal, with a higher transmission in the south than in the north and higher levels during the rainy season than during the dry season. In northern Senegal, despite a fairly desert-like environment, the presence of flooding rivers during the rainy season, as well as irrigation practices and seasonal migration, contribute to the transmission of the parasite. However, the transmission remains very low in the area of Richard Toll, averaging 1.2 cases per 1000 inhabitants (Diop, 2012). According to the 2008-2009 DHS data, the national prevalence of malaria at the time of the survey was 5.7%. The lowest levels were found in the region of Saint-Louis (the region where Richard Toll is located), where no cases were detected, and in the regions of Dakar (0.8%) and Louga (1.4%) (DHS, 2008). This success can be attributed to the strong involvement of local institutions, who provide excellent distribution of impregnated mosquito nets during prenatal consultations for women and enable access to effective antimalarials and rapid diagnostic tests, without supply disruptions (Diop, 2012). In our study, which will take place exclusively during the dry season, we can therefore reasonably not take into account the effect of malaria on anemia.

On the other hand, irrigation practices have drastically increased cases of schistosomiasis in the area of Richard Toll. However, as cases of schistosomiasis are most frequent among young boys who become infected while working in the fields, it is unlikely that our study population (children ages 2 to 5 years) is confronted with this parasitic disease. We can also ignore the effect of bilhiarzose in our study of anemia. Finally, for other common helminth infections in children (roundworm, hookworm, Trichinella), deworming with mebendazole is advocated for children over one year of age (WHO, 2003). The establishment of a mebendazole distribution during the first round of household data collection for children included in the study (if they did not receive treatment during the last 3 months) will allow for comparability of children concerning this factor, at the beginning of the study.

**10.3. First household data collection (Baseline)**

An initial survey of households will take place in January 2013 to gather baseline data. The questionnaire, in Fulani, will collect data related to:
- Composition of the household
- Herding/cows (size of herd, etc.)
- Crops (crop type, etc.)
- Socioeconomic status (household goods, level of education, etc.)
- Household food consumption (frequency, food security, etc.)

Additionally, measurements of hemoglobin levels will be conducted for children 6 months to 5 years, as well as their mothers, by drawing a drop of blood using a lancet and placing it in a microcuvette, allowing a direct reading of the result using a HemoCue® Analyzer

**10.4.** **Intervention**

The distribution phase of the incentive will last 6 months, from January 2013 to July 2013. The incentive of fortified Thiakry will be distributed daily by the milk collectors. Participants in the study who are part of the treatment group will have the daily opportunity to buy fortified Thiakry directly from the milk collection truck. During the study, if a herder responsible for the milk container has committed to providing a certain quantity of milk daily to LDB and does not honor their commitment one or two days in the same week, they will receive a warning (if they are in the treatment group). If the herder does not provide milk for 3 or more days in the week and is in the treatment group, they will not receive the fortified Thiakry the following week.

**10.5. Second household data collection**

A second phase of data collection will take place amongst households in July 2013, with a questionnaire and hemoglobin measures.

**10.6. Third household data collection**

A third phase of data collection will take place amongst households in January 2014, with a questionnaire and hemoglobin measures.

# 11. Biometrics and data analysis

**11.1. Results analysis plan**

A descriptive analysis will first be carried out, along with a comparison of baseline characteristics between both groups: proportion of children who are anemic, average hemoglobin levels, attrition of households etc. Covariates collected during the study at baseline (socio-economic level of the household, household composition etc.) corresponding to the two groups will be compared through univariate analysis. The efficacy of treatment will be evaluated using an adjusted multivariate analysis, adjusting using covariates that may have a confounding factor: length of participation in the study or covariates whose distribution is different between the 2 groups.

The analyses will be performed on the main response variable "proportions of households respecting the contract outlined at the beginning of the study", in terms of volume and milk collection frequency. These proportions will be compared between the two groups.

Secondary responses variables (proportion of children presenting mild, moderate or severe anemia; proportions of children with stunting, undernutrition or wasting) will be analyzed on the intent-to-treat population, gathering all the children who were attributed to a group for whom hemoglobin measurement results are available over 6 months, regardless of the group. The analysis will be adjusted for age and risk factors for anemia if their distributions are different between the two groups.

**11.2. Methods and data analysis models**

The impact of the intervention will be analyzed using the "difference in differences" method: the difference in the prevalence of anemia for children 2 to 5 years or the difference of average hemoglobin levels, i) between the treatment and the control group and ii) between the beginning and the end of the intervention. It will be possible to use a propensity score, determined from the baseline characteristics, in order to check the comparability between groups and, if necessary, to use the score as an adjustment factor. All tests performed will be unilateral, with a 95% confidence interval. The analysis will be performed using STATA 12.0 (Stata statistical software, Stata Corporation, College Station, Texas USA).

**11.3. Attrition**

Attrition will comprise households who have moved out of the area reached by collection routes and/or who have decided to go on transhumance and to leave the study voluntarily. The reasons for attrition will be recorded by enumerators. The characteristics of these households will be compared with those of households who participated all the way to the end of the study.

**11.4. Blinding**

As much as possible, the enumerators will not have knowledge of the intervention group at the time of the hemoglobin and anthropometric measures or at the time of questionnaire data collection.

# 12. Administrative procedures and considerations/Practical aspects

**12.1. Partners**

GRET will oversee the implementation of the study, recruitment of households, and random group assignment. An agreement between GRET, LDB, PPZS (Pastoralisme et Zones sèches en Afrique de l’Ouest), and IFPRI will be signed in order to share the data and human, financial, and logistical resources. This will ensure synergy in their actions, as they work together to promote the integration of the research results into future economic and social development initiatives and to share the results to meet the population’s evolving needs. Gaston Berger University will be responsible for the implementation of the data collection and CLM for the implementation of the awareness campaign.

**12. 2. Monitoring and data management**

The questionnaires will be entered directly on tablets (PDA) by enumerators recruited for data collection by Gaston Berger University (Saint-Louis). The data will then be processed anonymously (using unique identifiers).

**12.3. Ethical and regulatory aspects**

This study will be carried out in accordance with the research protocol; with the text of the Helsinki Declaration (adopted by the World Medical Assembly in June 1964 with amendments in Tokyo (1975), Venice (1983), Hong Kong (1989), Edinburgh (2000), Washington (2002), Tokyo (2004) and Seoul (2008)); and with the recommendations of good clinical practice (ICH, May 1996). It will also proceed in accordance with the laws and regulations of Senegal pertaining to matters of health research. This project was jointly submitted to the National Ethics Committee for Health Research in Senegal (CNERS) and the ethics committee of IFPRI.

**12.4. Information sharing, archiving, and document storage at the end of the study**

The partners have agreed not to share the raw data present in the databases. In particular:
- Trade secrets of LDB (preparation of recipes including Thiakry) cannot be shared. Only fortification levels and associated molecules may be detailed in research publications.
- Data collected daily by LDB will not be used for publications that will be made public within 6 months after the data’s collection.

- Data from household surveys conducted by IFPRI, GRET, and PPZS will not be disseminated in raw format without the agreement of the institution who collected them. In all cases, the last names, first names, geographical coordinates, and all other identifying information cannot be diffused to third parties.

- The source of all data should be clearly mentioned during oral communications (presentations) or written communications (publications).

The principal investigators and the coordinating center will archive and keep documents related to the study for a period of at least 30 years. These documents will include the protocol of the study and the informed consent of mothers, as well as any administrative documents.

**13. Publications**

The results of the research will be owned by IFPRI, which undertakes to disseminate them to LDB and the general public through technical fact sheets, articles in international journals, and communications during national and international meetings. Researchers involved in the project, regardless of nationality, will be cited in articles that come from the research. A final report will be delivered at the end of the study to different participating institutions, CLM, and the National Ethics Committee for Research in Health Senegal (CNERS).

**14. Financing**

Funding for the study comes from the European Union, through its CRP4 program "Agriculture for Nutrition and Health".

**15. Bibliography**

1. Arimond M, Wiesmann D, Becquey E, Carriquiry A, Daniels MC, Deitchler M, et al. Simple Food Group Diversity Indicators Predict Micronutrient Adequacy of Women ’ s Diets in 5 Diverse, Resource-Poor Settings. 2010;2059–69.

2. Berger J, Dillon J. Strategies de contrôle de la carence en fer dans les pays en developpement. 2002;22–30.

3. Berger J, Wieringa F, Lacroux A, Dijkhuizen M. Strategies to prevent iron deficiency and improve reproductive health. Nutr Rev. 2011;69 Suppl(1):S78–86.

4. Bhutta Z a, Ahmed T, Black RE, Cousens S, Dewey K, Giugliani E, et al. What works? Interventions for maternal and child undernutrition and survival. Lancet. 2008 Feb 2 [cited 2012 Oct 5];371(9610):417–40.

5. Black RE, Allen LH, Bhutta ZA, Caulfield LE, de Onis M, Ezzati M, Mathers C RJM and CUSG. Maternal and child undernutrition: global and regional exposures and health consequences. Lancet. 2008;371(9608):243–60.

6. Chang S, Huang Z, Ma Y, Piao J, Yang X, Zeder C, et al. Mixture of ferric sodium ethylenediaminetetraacetate (NaFeEDTA) and ferrous sulfate: an effective iron fortificant for complementary foods for young Chinese children. Food Nutr Bull. 2012;33(2):111–6.

7. CLM. Analyse de la situation nutritionnelle du Senegal, Enquête Smart Nationale. 2012.

8. De-Regil L, Suchdev P, Vist G, Walleser S, Pena-Rosas J, Collaboration TC. Home fortification of foods with multiple micronutrient powders for health and nutrition in children under two years of age ( Review ). 2011.

9. DHS. Senegal Enquête Demographique et de Sante à Indicateurs Multiples [Internet]. Dakar, Senegal; 2011. Available from: http://www.fao.org/docrep/013/i1683e/i1683e.pdf

10. DHS. Senegal Enquête nationale sur le paludisme (2008-2009) [Internet]. Available from: http://www.measuredhs.com/publications/publication-mis5-mis-final-reports.cfm

11. Diop C, Mutombo M. Fighting Malaria Together Newsletter. Senegal’s Richard Toll District leads the way in eliminating malaria [Internet]. 2012. Available from: http://www.fao.org/docrep/013/i1683e/i1683e.pdf

12. Eden A. Iron deficiency and impaired cognition in toddlers: an underestimated and undertreated problem. Paediatr Drugs. 2005;7(6):347–52.

13. FAO. The Contribution of Nutrition to Achieving the Millennium Development Goals [Internet]. Available from: http://www.fao.org/ag/agn/nutrition/Contribution of Nutrition to Achieving the Millennium Deve.pdf

14. FAO. The State of Food Insecurity in the World Addressing food insecurity in protracted crises [Internet]. 2010. Available from: http://www.fao.org/docrep/013/i1683e/i1683e.pdf

15. FAO/WHO. JOINT FAO / WHO EXPERT COMMITTEE ON FOOD ADDITIVES Sixty-eight meeting [Internet]. geneva; 2007 p. 1–18. Available from: http://www.fao.org/docrep/013/i1683e/i1683e.pdf

16. Gardner W, James R, Edgerton VR. Physical subjects work capacity and metabolic stress with iron deficiency. 1977;(June):910–7.

17. Gera. Effect of iron-fortified foods on hematologic and biological outcomes : systematic review of randomized controlled trials 1 – 4. 2012.

18. McLean E, Cogswell M, Egli I, Widjyla D, DeBenoist B. Worldwide prevalence of anaemia, WHO Vitamin and Mineral Nutrition Information System, 1993-2005. Public health nutrition. 2009 Apr p. 444–54.

19. McLean E, Cogswell M, Egli I, Wojdyla D, de Benoist B. Worldwide prevalence of anaemia, WHO Vitamin and Mineral Nutrition Information System, 1993-2005. Public health nutrition. 2009 Apr [cited 2012 Oct 8];12(4):444–54.

20. Micronutrients Initiative, COSFAM. Situation de base du statut en vitamine A, en Fer et en Zinc chez les enfants ages de 12-59 mois et chez les femmes en âge de procreer (15-49 ans) dans le cadre du programme de fortification des aliments en micronutriments au Senegal [Internet]. 2012. Available from: http://www.fao.org/docrep/013/i1683e/i1683e.pdf

21. Micronutrients Initiative, COSFAM. Situation de base du statut en vitamine A, en fer et en Zinc chez les enfants ages de 12-59 mois et chez les femmes en âge de procreer (15-49 ans) dans le cadre du programme de fortification des aliments en micronutriments au Senegal. 2012.

22. Olney DK, Rawat R, Ruel MT. Identifying Potential Programs and Platforms to Deliver Multiple Micronutrient Interventions 1, 2. 2012;

23. Parisse M. Enquête sur les fournisseurs de la Laiterie du Berger. 2011.

24. Staubli F, Adou P, Davisdsson L, Cook J, Hurrell R. Prevalence of iron deficiency with and without concurrent anemia in population groups with high prevalences of malaria and other infections: a study in Côte d  ’Ivoire. The American journal of Clinical Nutrition. 2001 p. 776–82.

25. Stoltzfus RJ, Chwaya HM, Montresor A, Albonico M, Savioli L, Tielsch JM. Community and International Nutrition Malaria , Hookworms and Recent Fever Are Related to Anemia and Iron Status Indicators in 0- to 5-y Old Zanzibari Children and These Relationships Change with Age 1. 2000;(January):1724–33.

26. Stoltzfus R. Iron deficiency: global prevalence and consequences. Food Nutr Bull. 2003;24:S99–103.

27. Talukder K. Using micronutrient malnutrition to take over food – the example of Shakti Doi in Bangladesh [Internet]. Available from: http://www.ibfanasia.org/gc/micronutrients-bangladesh-Dr.Khurshid-Talukder.pdf 28. Tolentino K, Friedman JF. An update on anemia in less developed countries. The American journal of tropical medicine and hygiene 2007 Jul;77(1):44–51.

29. Troesch B, van Struijvenberg M, Smuts C, Kruger H, Biebinger R, Hurrell R, et al. A Micronutrient Powder with Low Doses of Highly Absorbable Iron and Zinc Reduces Iron and Zinc Deficiency and Improves Weight-For-Age Z-scores in South African Children. 2011.

30. van Struijvenberg M, Smuts C, Lombard C, Dhansay M. Fortifying brown bread with sodium iron EDTA, ferrous fumarate, or electrolytic iron does not affect iron status in South African schoolchildren. The Journal of nutrition. 2008 Apr p. 782–6.

31. Viteri F. The Consequences of Iron Deficiency and Anaemia in Pregnancy on Maternal Health, the Foetus and the Infant. Adv Exp Med Biol. 1994;352:127–39.

32. Walker SP, Wachs TD, Gardner JM, Lozoff B, Wasserman GA, Pollitt E, et al. Child development in developing countries 2 Child development : risk factors for adverse outcomes in developing countries. 2007;145–57.

33. WHO. Controlling disease due to helminth infections [Internet]. 2003. Available from: http://www.fao.org/docrep/013/i1683e/i1683e.pdf

34. WHO/FAO. Guidelines on food fortification with micronutrients [Internet]. 2006. Available from: http://www.fao.org/docrep/013/i1683e/i1683e.pdf

35. WHO/Unicef. Focusing on anaemia: Towards an integrated approach for effective anaemia control [Internet]. 2004 [cited 2012 Sep 10]. Available from: http://whqlibdoc.who.int/hq/2004/anaemiastatement.pdf

36. Yang Z, Siekmann J, Schofield D. Fortifying complementary foods with NaFeEDTA--considerations for developing countries. Maternal & child nutrition. 2011 Oct p. 123–8.

1. A "Value Chain" is a supply chain in which value is added to a product as it advances along the chain. It is described by the series of activities and actors involved along the value chain and how and when the value is added by these actors (Hawkes & Ruel, 2010). The supply chain includes all operations beginning with inputs such as raw materials and incorporating all the changes required to achieve the final product. It is based on a contractual relationship between the actors. [↑](#footnote-ref-1)
