# Supplementary material for: Delivery of iron-fortified yoghurt, through a dairy value chain program, increases hemoglobin concentration among children 24 to 59 months old in Northern Senegal: A cluster-randomized control trial
Source: PLoS One. 2017 Feb 28;12(2):e0172198. doi: 10.1371/journal.pone.0172198 (PMC5330480; doi:10.1371/journal.pone.0172198)
Supplement: S1 Table — (DOCX) [file pone.0172198.s003.docx]

**S3 Table: Household level of exposure (children in the household consuming MNFY during the 3 days prior to the survey) and children living in these HH, in the intervention group, all children, boys and girls, 24 to 59 months of age**

|  | HH level | | | | Children level | | | | | | | | |  |
| --- | --- | --- | --- | --- | --- | --- | --- | --- | --- | --- | --- | --- | --- | --- |
|  | HHs surveyed in the intervention group | HHs with questionnaire on exposure | HHs having children consumingMNFY 3 days prior survey | | All | | | Boys | | | Girls | | |  |
|  |  |  |  |  | Total number of children of target age | Children of target age living in a HH receiving MNFY | | Total number of children of target age | Children of target age living in a HH receiving MNFY | | Total number of children of target age | Children of target age living in a HH receiving MNFY | |  |
|  | N | N | N | % | N | N | % | N | N | % | N | N | % | p |
|  |  |  |  |  |  |  |  |  |  |  |  |  |  |  |
| F1 | 116 | 113 | 71 | 63 | 158 | 100 | 63 | 80 | 53 | 66 | 78 | 47 | 60 | 0.57 |
|  |  |  |  |  |  |  |  |  |  |  |  |  |  |  |
|  |  |  |  |  |  |  |  |  |  |  |  |  |  |  |
| F2 | 126 | 123 | 107 | 87 | 163 | 139 | 85 | 78 | 67 | 86 | 85 | 72 | 85 | 0.87 |
|  |  |  |  |  |  |  |  |  |  |  |  |  |  |  |
| Endline | 132 | 131 | 79 | 60 | 190 | 118 | 62 | 95 | 58 | 61 | 95 | 60 | 63 | 0.83 |

Comparison of percentage of children of target age living in HH receiving MNFY, between boys and girls. P-values obtained with mixed linear regression testing the difference between genders, controlling for clustering (random effect) at the concession level. Values are N and percentages. F1: follow-up survey 1; F2: follow-up survey 2; HH: household; MNFY: micronutrient fortified yoghurt.
