# Supplementary material for: Delivery of iron-fortified yoghurt, through a dairy value chain program, increases hemoglobin concentration among children 24 to 59 months old in Northern Senegal: A cluster-randomized control trial
Source: PLoS One. 2017 Feb 28;12(2):e0172198. doi: 10.1371/journal.pone.0172198 (PMC5330480; doi:10.1371/journal.pone.0172198)
Supplement: S2 Table — (DOCX) [file pone.0172198.s004.docx]

**S4Table. Children surveyed by intervention group at different time points, all children 24 to 59 months of age, boys and girls**

|  | All | | All | | | |  | Boys | | | |  | Girls | | | |  | Comparison  boys vs girls (all) | Comparison  boys vs girls in intervention group | Comparison  boys vs girls in control  group |
| --- | --- | --- | --- | --- | --- | --- | --- | --- | --- | --- | --- | --- | --- | --- | --- | --- | --- | --- | --- | --- |
|  |  |  | Intervention | | Control | |  | Intervention | | Control | |  | Intervention | | Control | |  |  |  |  |
|  |  |  |  |  |  |  |  |  |  |  |  |  |  |  |  |  |  |  |  |  |
| **Presence during surveys** | N | % | N | % | N | % | p | N | % | N | % | p | N | % | N | % | p | p | p | p |
|  |  |  |  |  |  |  |  |  |  |  |  |  |  |  |  |  |  |  |  |  |
| Baseline | 449 | 100 | 204 | 100 | 245 | 100 |  | 102 | 100 | 129 | 100 |  | 102 | 100 | 116 | 100 |  |  |  |  |
|  |  |  |  |  |  |  |  |  |  |  |  |  |  |  |  |  |  |  |  |  |
| F1 | 370 | 82 | 170 | 83 | 200 | 82 | 0.97 | 89 | 87 | 108 | 84 | 0.91 | 81 | 79 | 92 | 79 | 0.96 | 0.27 | 0.85 | 0.34 |
|  |  |  |  |  |  |  |  |  |  |  |  |  |  |  |  |  |  |  |  |  |
| F2 | 376 | 84 | 171 | 84 | 205 | 84 | 0.89 | 83 | 81 | 104 | 81 | 0.75 | 88 | 86 | 101 | 87 | 0.87 | 0.12 | 0.34 | 0.26 |
|  |  |  |  |  |  |  |  |  |  |  |  |  |  |  |  |  |  |  |  |  |
| Endline | 423 | 94 | 191 | 94 | 232 | 95 | 0.63 | 95 | 93 | 122 | 94 | 0.88 | 96 | 94 | 110 | 95 | 0.60 | 0.88 | 0.77 | 0.93 |
|  |  |  |  |  |  |  |  |  |  |  |  |  |  |  |  |  |  |  |  |  |

Comparison of percentage of children surveyed between boys and girls in both group, in intervention group and in control group. P-values obtained with mixed linear regression testing the difference between study groups, and gender, controlling for clustering (random effect) at the concession level. Values are N and percentages. F1: follow-up survey 1; F2: follow-up survey 2
