# Supplementary material for: Delivery of iron-fortified yoghurt, through a dairy value chain program, increases hemoglobin concentration among children 24 to 59 months old in Northern Senegal: A cluster-randomized control trial
Source: PLoS One. 2017 Feb 28;12(2):e0172198. doi: 10.1371/journal.pone.0172198 (PMC5330480; doi:10.1371/journal.pone.0172198)
Supplement: S3 Table — (DOCX) [file pone.0172198.s005.docx]

**S5 Table. Impact of MNFY on prevalence of anemia (percentage points) among children 24 to 59 months of age with 4 measures, among all children, boys and girls (mixed models, random effect at cluster and individual level).**

|  | All | | Boys | | Girls | | All (anemia definition 10g/dL) | |
| --- | --- | --- | --- | --- | --- | --- | --- | --- |
| **N** | 449 |  | 231 |  | 218 |  | 449 |  |
|  | pp (95%CI) | p | pp (95%CI) | p | pp (95%CI) | p | pp (95%CI) | p |
| Intervention | -0.02 (-0.10;0.06) | 0.63 | -0.00 (-0.10;0.10) | 0.96 | -0.01 (-0.08;0.11) | 0.56 | 0.01 (-0.08;0.11) | 0.78 |
| Time (ref=Baseline) |  |  |  |  |  |  |  |  |
| F1 | -0.11 (-0.18;0.04) | <0.001 | -0.07 (-0.16;0.02) | 0.14 | -0.17 (-0.27; -0.06) | 0.01 | -0.09 (-0.16;-0.03) | <0.01 |
| F2 | -0.06 (-0.13;-0.01) | 0.07 | -0.06 (-0.15;0.04) | 0.16 | -0.07 (-0.17;0.03) | 0.17 | -0.15 (-0.22;-0.08) | <0.001 |
| Endline | -0.18 (-0.25;-0.11) | <0.001 | -0.18 (-0.27;-0.08) | <0.01 | -0.18 (-0.29;-0.08) | <0.01 | -0.23 (-0.30;-0.16) | <0.001 |
| Intervention x Time (ref=control & baseline) |  |  |  |  |  |  |  |  |
| Intervention x F1 | 0.06 (-0.05;0.16) | 0.27 | 0.00 (-0.13;0.14) | 0.98 | 0.12 (-0.03;0.28) | 0.12 | -0.06 (-0.17;0.04) | 0.21 |
| Intervention x F2 | -0.05 (-0.15;0.05) | 0.35 | -0.10 (-0.25;0.04) | 0.16 | 0.00 (-0.15;0.16) | 0.95 | -0.04 (-0.14;0.06) | 0.47 |
| Intervention x Endline | -0.02 (-0.12;0.08) | 0.72 | -0.04 (-0.18;0.10) | 0.60 | 0.00 (-0.16;0.16) | 0.99 | -0.09 (-0.19;0.01) | 0.07 |
|  |  |  |  |  |  |  |  |  |
| Child’s age at baseline | -0.00 (-0.01;-0.00) | <0.01 | -0.00 (-0.01;-0.00) | <0.01 | -0.0 (-0.01;0.00) | 0.07 | -0.00 (-0.01;-0.00) | <0.01 |
| Boys | 0.06 (0.00;0.11) | 0.03 | - |  | - |  | 0.08 (0.02;0.14) | 0.01 |
| Iron treatment at baseline | 0.20 (0.08;0.31) | <0.01 | 0.15 (0.00;0.30) | 0.05 | 0.14 (0.05;0.32) | 0.15 | 0.26 (0.12;0.40) | <0.001 |
| Wealth Index | -0.03 (-0.19;0.12) | 0.65 | 0.02 (-0.16;0.21) | 0.79 | -0.10 (-0.36;0.13) | 0.40 | -0.09 (-0.27;0.10) | 0.36 |
| Mother’s age | -0.00 (-0.01;-0.00) | 0.02 | -0.00 (-0.01;0.00) | <0.01 | -0.00 (0.76;0.00) | 0.59 | -0.00 (-0.01;0.00) | 0.02 |

Only adjusted models on covariates are presented (coefficient not varying in non-adjusted models). P-values obtained with mixed regression models, using random effect at child-level to take into account correlations between repeated measures (unstructured matrix of variance-covariance) and random effect at the concession-level (cluster), testing the interactions intervention*time. Values are percentage points (95%CI). F1: follow-up survey 1; F2: follow-up survey 2; Hb: hemoglobin; MNFY: micronutrient fortified yogurt
